# Farnesol-Like Endogenous Sesquiterpenoids in Vertebrates: The Probable but Overlooked Functional “Inbrome” Anti-Aging Counterpart of Juvenile Hormone of Insects?

**DOI:** 10.3389/fendo.2014.00222

**Published:** 2015-01-06

**Authors:** Arnold De Loof, Elisabeth Marchal, Crisalejandra Rivera-Perez, Fernando G. Noriega, Liliane Schoofs

**Affiliations:** ^1^Functional Genomics and Proteomics Group, Department of Biology, KU Leuven, Leuven, Belgium; ^2^Molecular Developmental Physiology and Signal Transduction Group, Department of Biology, KU Leuven, Leuven, Belgium; ^3^Department of Biological Sciences, Florida International University, Miami, FL, USA

**Keywords:** farnesol, insect hormones, sex steroids, development, puberty, Alzheimer, *Caenorhabditis elegans*, obesity

## Abstract

Literature on the question whether the juvenile stage of vertebrates is hormonally regulated is scarce. It seems to be intuitively assumed that this stage of development is automated, and does not require any specific hormone(s). Such reasoning mimics the state of affairs in insects until it was shown that surgical removal of a tiny pair of glands in the head, the *corpora allata*, ended larval life and initiated metamorphosis. Decades later, the responsible hormone was found and named “juvenile hormone” (JH) because when present, it makes a larva molt into another larval stage. JH is a simple ester of farnesol, a sesquiterpenoid present in all eukaryotes. Whereas vertebrates do not have an anatomical counterpart of the *corpora allata*, their tissues do contain farnesol-like sesquiterpenoids (FLS). Some display typical JH activity when tested in appropriate insect bioassays. Some FLS are intermediates in the biosynthetic pathway of cholesterol, a compound that insects and nematodes (=Ecdysozoa) cannot synthesize by themselves. They ingest it as a vitamin. Until a recent (2014) reexamination of the basic principle underlying insect metamorphosis, it had been completely overlooked that the Ca^2+^-pump (SERCA) blocker thapsigargin is a sesquiterpenoid that mimics the absence of JH in inducing apoptosis. In our opinion, being in the juvenile state is primarily controlled by endogenous FLS that participate in controlling the activity of Ca^2+^-ATPases in the sarco(endo)plasmic reticulum (SERCAs), not only in insects but in all eukaryotes. Understanding the control mechanisms of being in the juvenile state may boost research not only in developmental biology in general, but also in diseases that develop after the juvenile stage, e.g., Alzheimer’s disease. It may also help to better understand some of the causes of obesity, a syndrome that holometabolous last larval insects severely suffer from, and for which they found a very drastic but efficient solution, namely metamorphosis.

## Introduction

**Figure 1:**
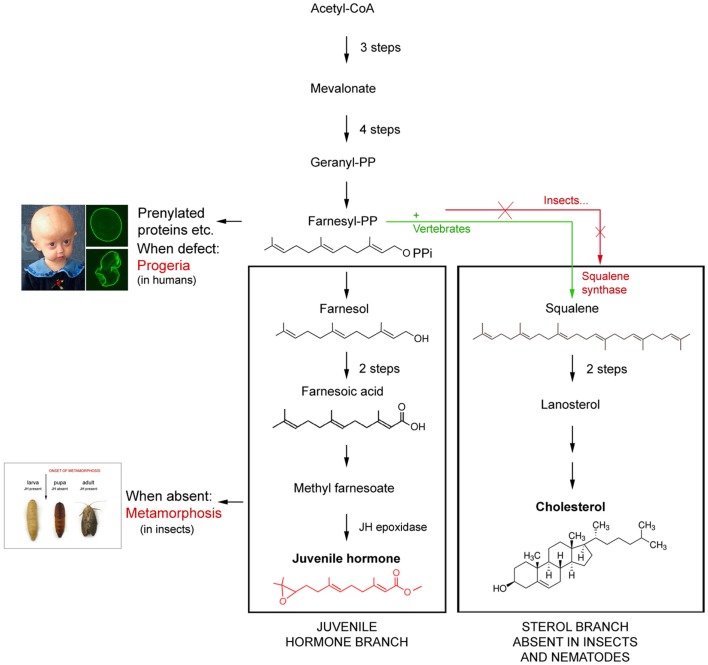
**Schematic representation of the difference in the mevalonate-based biosynthetic pathway in insects and nematodes (=Ecdysozoa) on one hand, and vertebrates on the other**. The main difference is that Ecdysozoa cannot synthesize cholesterol on their own because they miss the key enzyme squalene synthase. Whether their common ancestor lost the coding gene or never had it remains a matter of discussion. Modified after De Loof et al. (2).

All animals, plants and fungi pass through a juvenile stage before becoming reproductively active and next becoming aged. In holometabolous insects a high titer of juvenile hormone (JH), an ester of the endogenous sesquiterpenoid alcohol farnesol (Figure 1) keeps larvae in their juvenile state. JH can occur is several isoforms^1^ (1). It makes a larva molt into another larval stage. This is known as the “*status quo* effect” of JH. When the JH titer drops to zero at the end of larval life, metamorphosis is initiated. When metamorphosis nears completion, the JH titer rises again and the enclosing adult enters or completes (if the gonads started developing already during metamorphosis) the reproductive phase, and concurrently ages rapidly.

Hitherto, it was assumed that vertebrates do not have such a juvenilizing hormone, or, if they do have one, thyroid hormones, in particular triiodothyronine (T3) is likely candidates. Like the absence of JH has a key role in initiating metamorphosis in all holometabolous insects, the presence of T3 has a prominent role in initiating metamorphosis in some fish and amphibian species (3). Remarkably T3 has been shown to be active in some insect bioassays (4, 5). However, functional evidence supporting this view remains unconvincing (2). In particular the lack of a suitable bioassay(s) which is indispensable for monitoring the successive steps in the purification procedure represents the bottleneck for identifying the JH of vertebrates, if it exists. As long as this situation continues, one has no choice but exploring the possibilities of comparative endocrinology between vertebrates and invertebrates for finding an alternative strategy for unraveling the control mechanisms for initiating and maintaining the juvenile state in vertebrates.

The leading idea in this paper is that being in the juvenile state as an animal, follows from controlling principles that were already present in the common ancestor of plants, fungi, protostomian, and deuterostomian animals. Hence the signaling pathways involved are probably largely over a billion years old. It is therefore more likely that their key components have been well conserved in evolution, rather than that deuterostomes (to which vertebrates belong) “all of a sudden” invented a completely new strategy for generating and temporarily maintaining a juvenile state. It is well-known that insects, which are protostomes, use JH as their juvenilizing agent. Hence, the question is: could it be that in vertebrates, and by extension in all deuterostomes, the basic elements of this endogenous sesquiterpenoid system continue to be present and operate in a similar way, perhaps in a slightly modified form?

In this concept paper, we will argue that this is probably the case. We will also outline that research into aging-related diseases may benefit from the novel insights.

## Wording in Short of the Paradigm

Both deuterostomes and protostomes must have inherited the basic principles of passing through a juvenile state before becoming sexually mature from their common ancestor. These principles are probably still operational in both. Insects, in particular holometabolous ones which in this respect are best studied, use JHs as their key hormone for realizing and maintaining the juvenile state. The Ca^2+^-ATPase present in the sarco(endo)plasmic reticulum (SERCA) likely functions as the largely overlooked membrane receptor for JH and for at least some other endogenous farnesol-like sesquiterpenoids (FLS). No functional hormonal counterpart of JH has yet been identified in any vertebrate. Like all eukaryotes, vertebrates do have farnesol and some other FLS, but their role is only partially understood. We think that being a juvenile vertebrate depends upon the interaction of FLS with the Ca^2+^-homeostasis system, like this is the case in insects. However, the key difference with insects is that in vertebrates farnesol/FLS is probably not a hormone. Instead, its precursor farnesyl-pyrophosphate (FPP) is present and probably synthesized in all cells of the body as one of the precursors in the cholesterol biosynthetic pathway. Because insects and nematodes either lost or never had the gene coding for the enzyme squalene synthase that converts squalene (that is formed from FPP) into cholesterol, they cannot make cholesterol by themselves. They solved this problem by ingesting cholesterol as a vitamin and by using a farnesol ester, namely JH as a circulating hormone to control at least one of the components of the Ca^2+^-homeostasis system, namely the SERCA-pump system.

## Constructing the Paradigm

### How is the juvenile state defined?

A juvenile is an individual organism that has not yet reached its adult form, sexual maturity or size. In particular in holometabolous insects, the juvenile state corresponds to all larval stages before metamorphosis is initiated. In amniote vertebrates, the embryo represents the larval stage, and “juvenile” applies to the time between hatching/birth and reaching maturity.

Animals in which the transition from larva/juvenile to sexually mature adult takes only days (most insects) are better experimental models for studying the principles of being a juvenile than the ones in which this transition takes months to even years.

#### Extracts of mammalian tissues do contain “JH bioactivity”

In the pioneering decades (1960–1980) following the identification of the chemical nature of the two non-peptidergic key insect hormones, JH, and the steroid molting hormone ecdysone, various tissues from insects but also from other species, including mammals, were analyzed for the presence of compounds with JH-like activity in specific insect bioassays. Some remarkable results were obtained. In insects the most unexpected one was that not the *corpora allata*, tiny glands in the head thought to be the only site of JH synthesis, were the richest source of JH, but the adult male accessory glands of the moth *Hyalophora cecropia*, an important experimental model at that time. The huge JH concentrations present in these glands which are the physiological counterpart of the prostate gland of mammals enabled the purification of JH. It turned out to be an ester of farnesol. At that time, FPP was already known as a precursor of cholesterol in vertebrates. The fact that insects cannot synthesize cholesterol by themselves made researchers logically conclude that farnesol/FLS in insects only served as the precursor for JH. A second remarkable result was that Williams et al. (6) showed that extracts from several mammalian tissues (thymus, human placenta, and others) displayed activity when tested in typical bioassays for insect JH.

Analysis of the chemical nature of the JH-bioactive substance in extracts of mammalian tissues showed that the active factor was farnesol, the same as the active factor present in excrements of the mealworm *Tenebrio molitor* and in yeast (7, 8). Thus vertebrates do have farnesol, and this farnesol, along with other compounds, some of them being farnesol-like (9) is active in insect JH-bioassays. Yet, although this finding was intriguing, it was not further analyzed in depth because it seemed normal that a direct precursor of JH, of which the chemical identity was elucidated by Röller and Dahm (10), could also have moderate bioactivity. The data got forgotten because it was deemed irrelevant for vertebrate physiology. Moreover, one should keep in mind that in those days, the idea that the endocrine systems of vertebrates and invertebrates might have some elements in common, was far from readily accepted. To date, farnesol is best known in the perfume industry where it is used as an adjuvant. It was named after the Farnese acacia tree (*Vachellia farnesiana*) of which the flowers are a rich source of farnesol. Its exact role in plants remains to be elucidated.

### Little progress in the past 45 years

To date, we know that the basic principles of the endocrine systems of both vertebrates and insects are very similar, probably because they were already present in their common ancestor. We also have a good idea which mutations resulted in differences in their steroids, endogenous sesquiterpenoids, and peptide hormones (11, 12). Current vertebrates no longer have the genes/enzymes needed for synthesizing ecdysteroids which are probably more “ancient” than the vertebrate-type steroids. On the other hand, insects either never had (2, 13) or lost the enzymes needed for biosynthesizing most “modern vertebrate-type steroids”. Yet, some such “vertebrate-type” steroids like pregnenolone, testosterone, eestradiol, etc. are present in some insect species, but their function, if any, has not yet been found (14, 15). The biosynthetic pathway of FLS is well known (16) (Figure 1). However, the exact mode of action of JH is still a matter of discussion (2, 17–19).

### Never total absence of farnesol in the life cycle of vertebrates

In insects there is a natural condition in which JH is totally absent, namely during metamorphosis of holometabolous insects. When synthetic JH is applied before metamorphosis is initiated, larval life is usually extended. Depending upon the species, one or more extra larval instars can be formed, proving that JH is indeed a hormone that maintains the juvenile state.

In vertebrates, the situation “zero farnesol/FLS,” a necessary tool for defining the role of farnesol is never realized during lifetime. This is due to the fact that unlike in insects where – in larvae – farnesol and JH are only synthesized in the *corpora allata*, FLS are present in all cells of the vertebrate body (Table 1, see later). This is probably the case throughout all developmental stages.

**Table 1:** **Occurrence of farnesol, its precursor farnesyl pyrophosphate (FPP) and its derivatives, farnesal and farnesoic acid in a variety of tissues of a male mouse (*Mus musculus*)**.

	FPP	Farnesol	Farnesal	FA
Brain	**+**	**−**	+	**+**
Muscle	**−**	+	+	+
Thymus	**−**	+	+	+
Salivary gland	**−**	+	+	**+**
Gut	**−**	+	+	**+**
Liver	**−**	**−**	**+**	+
Testis	+	+	+	**+**
Prostate	+	+	+	+
Blood	+	+	+	+

*Metabolites were extracted from about 20 mg of tissue and 5 μl of blood*.

*FPP, farnesyl pyrophosphate; FA, farnesoic acid*.

*Meaning of other symbols: compared to values present the *corpora allata* of insects, the best studied tissue in this respect: −: close to the detection limit; **+**: relatively high concentrations; +: moderate concentrations*.

In vertebrate physiology, the question whether, perhaps, farnesol/FLS may have a function on its own, additional to that of FPP in prenylation and in serving as a precursor of cholesterol has not (yet) been an issue.

### Total absence of JH in insects mimics some of the effects of the SERCA-pump blocker thapsigargin

What happens in insects when the JH/farnesol titer drops to zero? In holometabolous insects total absence of the sesquiterpenoid JH is a regular part of the life cycle. Indeed, the *corpora allata*, the only site of farnesol and JH synthesis in juvenile insects become completely inactive at the end of larval life. Furthermore, all JH that still circulates in the hemolymph is also fully degraded by specific esterases. This results in the zero JH/FLS situation. Exactly this absence of JH rather than the release of the prothoracicotropic hormone (13) initiates metamorphosis. The most dramatic aspect of metamorphosis is the programed cell death/apoptosis of the tissues that actively secrete proteins such as the fat body, the alimentary canal, the salivary- and prothoracic glands, etc. (2).

What happens in vertebrates when an endogenous FLS can no longer exert its normal function becomes apparent from the effects of administration of the plant toxin thapsigargin, which is, like farnesol/JH also a sesquiterpenoid. Thapsigargin blocks the SERCAs of both vertebrates and insects. This indicates that not only the overall structure of SERCAs has been very well conserved in evolution, but that the binding site of thapsigargin on the SERCAs has also been conserved. De Loof (20) argued that this binding site of sesquiterpenoids on the endoplasmic reticulum functions as a third type/family of receptors that complements the much better documented plasma membrane receptors and nuclear receptors for (some types) of hormonal ligands.

In thapsigargin-sensitive cells both the absence of JH as well as the administration of thapsigargin make the SERCAS stop pumping Ca^2+^ into the lumen of the smooth- and rough endoplasmic reticulum (SER/RER). As a result the Ca^2+^-concentration in the cytosol increases up to the level that Ca^2+^-induced apoptosis (21) is induced.

### Why did nematodes, in particular *Caenorhabditis elegans*, retain the genes for synthesizing farnesol during hundreds of millions years?

Like in insects, the enzyme squalene synthase which is essential for the biosynthesis of cholesterol is absent in nematodes. Either these Ecdysozoa lost the gene or they never had it (20). This situation probably dates from before the divergence of deuterostomian and protostomian animals, or, perhaps from even before the divergence of plants, fungi, and animals, thus from at least one billion years ago. Yet, nematodes still express the genes coding for the enzymes that are instrumental to the biosynthesis of farnesol, its precursors and some metabolites, but not of cholesterol (22, 23). The fact that these genes have been conserved for so long indicates that farnesol/FLS must have a function different from that of merely serving a role in the biosynthesis of cholesterol; otherwise the genes would have become redundant. In worm research, this question triggered some interest, but for lack of performing quantification methods of FLS in small samples, a conclusive answer has not yet been given. What could that missing function be? The comparative approach with insects may provide the answer.

We performed an orienting experiment with the method of Rivera-Perez et al. (24) which will be outlined next. The results were that all FLS listed in Table 1 are present in juvenile and adult *C. elegans*. In due time, they will be published elsewhere.

### A novel, HPLC-based method for quantifying farnesol and its precursors has recently been described

The ancient data from Williams et al. (6) and of Schmialek (7) on the presence of farnesol in a few tissues of mammals did not give any indication on its origin, either from the ingested food or from an endogenous site of synthesis. All cells of the animal body need cholesterol. A commonly held view (25) is that the few major production sites of cholesterol, namely the liver and the intestine, secrete enough cholesterol into the bloodstream to comfort all peripheral tissues. In this scenario, one should not find the typical precursors and some metabolites of farnesol in these peripheral tissues. The other possibility is that all tissues express the genes for biosynthesizing farnesol, its precursors and some derivatives, not so much because they would not receive sufficient amounts of cholesterol from the blood and thus to compensate for such theoretical insufficiency, but because they all need farnesol/FLS for another reason than for using it for cholesterol synthesis.

Sesquiterpenoids are notoriously difficult to quantify accurately and robustly because of their lipophilic and labile nature and their tendency to bind non-specifically. Mass spectrometry approaches could be accurate, but are expensive and complex to interpret (1, 24).

Fortunately, a novel, very sensitive method developed by Rivera-Perez et al. (24) allows analyzing the whole biosynthetic pathway, from mevalonate up to farnesol and some derivatives (further to JH if needed) at once. The assay is based on the derivatization of analytes with fluorescent tags, with subsequent analysis by reverse phase HPLC coupled to a fluorescent detector (HPLC-FD).

### Presence of FRS in mammalian tissues

To verify if the old data of Williams et al. (6) are correct, we performed an orienting experiment with the Rivera-Perez et al. (24) method using extracts of tissues of a male mouse (*Mus musculus*). Although the method is able to generate both qualitative and quantitative data, we have limited ourselves to the qualitative ones. The reason is that the imposed deadline for submission of this paper did not allow generating a sufficient number of replicate experiments for an accurate quantification of all intermediates. Nevertheless, the results were convincing enough to state that all analyzed tissues of a male adult mouse seem to have different metabolites in the FLS pathway, and by extrapolation the different necessary enzymes for biosynthesizing several FLS, as shown in Table 1.

Contrary to what could be expected, FPP the direct precursor in the biosynthesis of squalene is only present in high concentrations in brain tissue and less in tissues known to be actively synthesizing cholesterol. FPP is also important for prenylation. The presence of FPP, farnesol, farnesal, and farnesoic acid in blood indicates that a role as a hormone may not *a priori* be ruled out, on condition that none originates from the food. In the literature there are no data suggesting a hormonal role. The high concentrations of farnesoic acid are intriguing. Farnesol and farnesal are quite toxic molecules in living cells. Rizzo and Craft (26) who studied the Sjögren–Larsson syndrome, demonstrated that conversion of farnesal to farnesol by an aldoketo reductase reduces the toxicity. The produced farnesol leaks out of the cells. The same effect was observed in insects in the JH pathway; the excess of farnesal in the *corpora allata* is converted back to farnesol that can leak out of the gland (27).

These preliminary results urge for an in depth qualitative and quantitative study of the farnesol/FLS biosynthetic pathway in all tissues of the body throughout development.

### Inbrome signaling versus a role as a hormone

The term “inbrome” stands for “intramembrane signaling substance.” It was introduced by De Loof et al. (2) to denote and categorize chemicals that act in the plane of membranes by binding to receptors that are located in membranes, in particular in intracellular membranes. A typical example is the plant toxin thapsigargin that binds to the Ca^2+^-ATPases located in the sarco(endo)plasmic reticulum.

In the past neither farnesol nor any other FLS has been advanced as a hormone of vertebrates. The fact that all tissues seem to have the biosynthetic machinery for some FLS, suggests that if these compounds do have a role in controlling some cell-physiological process, e.g., Ca^2+^-homeostasis they can do so from within the cell, without the need of having to be secreted into the blood stream first. The fact that single-celled organisms, like yeast, produce sesquiterpenoids, including farnesol, via the mevalonate pathway (28) supports this view. The “inbrome model” may explain some aspects of the mode of action of lipophilic hormones, in particular of sex steroids and JH. There is a major problem with the widely accepted Karlson model of steroid hormone action (29). It says that a steroid (ecdysone in Karlson’s experiments) diffuses right through the plasma membrane “unnoticed” (=without changing the permeability or electrical properties of the plasma membrane), next through the cytoplasm where it binds to a cytoplasmic receptor (=a later finding), and that finally this complex passes the nuclear pore complexes into the nucleus and binds there to the promoter regions of specific genes. Very few researchers are aware of the fact that this model was not at all readily accepted at the time it was launched. To the contrary, it was heavily disputed for good reasons. The most convincing counterargument was that the so called “specific” hormone-induced puffs could as well be induced in the absence of any hormone by changing the ionic concentration of some inorganic ions in medium in which the salivary glands were incubated (2, 30).

Karlson’s model is too simple. It is much more probable that once a lipophilic signaling molecule enters a membrane, it can cause electrical changes at the level of the plasma membrane, and even more important, it will rapidly diffuse throughout the whole continuum of all interconnected intracellular membranes. Because the plasma membrane forms a continuum with the endoplasmic reticulum and the outer part of the nuclear envelope, this means that lipophilic signaling molecules are omnipresent in membranes. For figures illustrating this chain of events, see De Loof (20) and De Loof et al. (2). The action of steroid hormone-receptor complexes inside the nucleus, which has been thoroughly studied in the past decades, is only one of the aspects of steroid hormone action, not at all the full story.

## The Juvenile State Ends at Puberty: Why and How?

At puberty the juvenile state transits into the reproductive state. In vertebrates, increased GnRH secretion causes increased synthesis and release of gonadotropins. In endocrinology the general consensus is that the gonadotropins LH and FSH “stimulate” the production of sex-related steroids (progesterone, estrogens, androgens, etc.). However, another interpretation is possible. De Loof et al. (13) stated that the appearance of peak concentrations of ecdysteroids in the hemolymph of metamorphosing and prediapausing insects coincides with the programed cell death of several tissues, in particular those that actively secrete proteins during larval life. Therefore, the appearance of such steroid hormone peaks indicates that, somewhere in the body, some tissue(s) is undergoing apoptosis, e.g., the fat body of insects or the follicle cell layer surrounding growing oocytes. According to this view the primordial role of the gonadotropins FSH and LH is not the “stimulate” the ovarian follicle cells to engage in producing steroid sex hormones, but to force them to undergo apoptosis. This has also been suggested before in our concept that reproduction is in fact a strategy of the cellular defense system (31). Later, De Loof et al. (13) evaluated whether a similar mechanism with a key role for apoptosis might be operational in the context of the onset of insect metamorphosis which marks the end of the juvenile state.

From “puberty” on, egg production starts. Eggs accumulate yolk proteins, lipids, glycogen, and last but not least huge amounts of Ca^2+^. The liver of vertebrates and its counterpart in insects, the fat body, secrete lots of yolk protein precursors, vitellogenins, into the bloodstream under the influence of female sex steroids. Any protein secretion through the RER–Golgi system is accompanied by the secretion of Ca^2+^ (20). A typical example is the production of milk in mammals. The Ca^2+^-concentration in milk amounts to about 50 mM. This should be compared to the Ca^2+^ concentration in the cytosol of unstimulated cells in general, which is as low as 100 nM. The onset of puberty therefore involves a drastic change in Ca^2+^-homeostasis, in particular in females. These changes have to be controlled one way or another. The FLS-Ca^2+^ homeostasis system is a primordial candidate to play a role in this process.

## The Possibility That Some SERCA Pump Isoforms Act as Promiscuous Receptors

In endocrinology, the consensus is that a ligand should bind with high affinity and specificity to a receptor. This holds true for plasma membrane receptors of the G-protein coupled type (GPCRs), and probably for intranuclear receptors as well as. We doubt that it also applies to lipophilic steroids and other lipophilic signaling substances like JH.

The question is raised whether the end of the juvenile state is caused by increasing concentrations of steroid hormones in the intracellular membrane continuum of cells that compete with FLS for binding to the receptor site on (some isoforms of) the SERCAs? In other words, does the endocrinological explanation for ending the juvenile state reside in the fact that the receptor site for farnesol/FLS on the SERCAs is (very?) promiscuous?

A major argument in favor of this view is that about 4,200 compounds have been synthesized or extracted from natural sources that are all active in bioassays for JH [=Juvenile Hormone Analogs or JHAs; (8)]. Some are farnesol-like; others have very different chemical structures. The most bioactive ones are synthetic peptides that are selectively active in insect species belonging to the Pyrrhocoridae. There are also anti-juvenile compounds (32), e.g., some benzodioxoles that act as non-mutagenic insect chemosterilants. It is improbable that all these compounds would bind to a different nuclear receptor but nevertheless generate similar morphological and physiological effects. The reason is that the number of nuclear receptors is estimated to be around 1,000. We think that it is more probable that many/most JHAs and sex steroids bind with a different affinity to a promiscuous receptor residing in the SER/RER. For other arguments, both in favor and against this view see De Loof (20).

Males and females do not have different sex steroids. In vertebrates both have estrogens and androgens. In insects both have 20-OH-ecdysone (=estrogen counterpart) and ecdysone (=testosterone counterpart) (20, 33). This raises the question which signaling system can handle a balance of hormones? We think that a promiscuous receptor can do so, although other possibilities cannot be excluded (20). Such a system takes into account not only the affinity of the ligand but the concentration of other competing ligands as well. A physiological effect is generated when the balance is “right.”

## Severe Obesity in Insects Near the End of the Juvenile State: A Causal Link with FLS/Ca^2+^-Homeostasis?

The juvenile state is characterized by fast growth. This requires that tissues deposit more proteins than lipids. At the end of the end of the juvenile state, this situation is reversed in most species. Storing more lipids is often considered as beneficial “because accumulating nutrient reserves will facilitate the growth of reproductive organs and production of gametes, etc.” This reasoning is incorrect because it is teleological. Individual tissues, such as adipose tissue, do not plan for the future at all. They do not deposit lipids and glycogen because that would be beneficial for the fitness of the whole organism, but because a signal inside the adipose tissue is causal to the increased lipid production and accumulation. The raises the question as to the nature of that signal. Systems in which such drastic accumulation occurs in a short time are good experimental models, e.g., during pre-metamorphosis and during pre-diapause in insects.

The literature on obesity in vertebrates, in particular in humans is truly vast. Indeed, a Pubmed search with “obesity” as query (05/10/2014) yielded 205,506 references. Yet, one cannot escape the impression that, despite the enormous research effort, there is still no consensus about the primordial cause(s). Maybe something has been overlooked. Could it be FLS/Ca^2+^-homeostasis?

The query “obesity and calcium” yielded 3,155 references while “obesity and farnesol” resulted in only 3 refs of which one was quite interesting. Duncan and Archer (34) administered farnesol to rats. They found that oral administration lowers serum triglyceride levels. They think that the effect is mediated by the downregulation of retinoid X receptor beta. These authors assumed that farnesol, as a dietary component, could play a role in lipogenesis and fatty acid oxidation, both impaired in obesity. The possibility that farnesol/FLS are secreted into the bloodstream from tissues that produce large amounts of farnesol/FLS and cholesterol was not considered. Our data in Table 1 show that one has to consider the possibility that FLS present in various tissues and in blood are synthesized by the body itself.

### Metamorphosis

We think that the FLS-lipogenesis connection deserves an in depth analysis. For this view, we focus on the situation in holometabolous insects like flies, beetles, butterflies, moths, etc. They differ from hemimetabolous ones like cockroaches, crickets, etc. in that they undergo a complete metamorphosis. The larval stage molts into a pupa and next into an adult. Prior to entering the pupal stage, the pre-metamorphic larvae undergo drastic changes. One in particular is that the penultimate and early last larval instars eat voraciously. A result is that their fat body accumulates huge amounts of lipids, glycogen, and some proteins as well with a fast gain in weight as a result. This eating comes to a complete stop during the last larval instar. Next they enter the wandering stage during which they leave the food in search for a suitable place to molt into a pupa. They empty their gut, by vomiting and/or defecation. In some species like, e.g., silk moths in which the silk glands are modified salivary glands, or *Drosophila* that uses secretions from their salivary glands as a glue to adhere to a substrate, these parts of the alimentary canal are also emptied. The larvae will become more and more immobile: they give the impression to enter in a coma-like state which lasts for some time. Meanwhile, the body is drastically reshaped. One of the changes is that the fat body is almost completely lysed. Like most other tissues that actively secreted proteins during the larval stage (2), they are replaced by adult-type tissues. Also the pupal cuticle is replaced by an adult one. At eclosion, a drastically “novel” organism makes its entrance into the world. Its larval morbid obesity is completely cured. Concurrently, the alimentary canal of the adult is drastically reshaped. Adults can still can ingest food (not the case anymore in some species that do no longer have mouthparts), but the food they can handle can be totally different from what they ate as larvae (e.g., in flies and in butterflies and moths; some beetle species do not change their diet). One could say that they underwent a severe but efficient bypass of the stomach in combination with adaptation to a novel diet.

For physiologists and endocrinologists, the interesting question is: What causes this larval obesity and how is it cured? The key endocrinological event/change that takes place at the end of larval life is that the production of JH by the CA comes to a complete standstill. Also, all JH that circulates in the hemolymph is completely metabolized by specific esterases. Therefore, it is likely that an important cause of aggravating obesity is the drop to zero of the JH titer. This is evidenced by the fact that application of synthetic exogenous JH can overrule the morbid obesity syndrome. This treatment induces, in some species at least, a larval molt into a supernumerary instar (8).

### Preparing for diapause

Accumulation of lipids in the fat body in the last larval instar is not just a matter of more lipids that are deposited. In the absence of JH, the whole physiology of the fat body changes. This has been shown long ago by De Loof and Lagasse (35). They studied the changes in ultrastructure of the fat body of adult Colorado potato beetles raised in two different photo-regimes. Beetles raised in long day conditions (more than 12 hr light per day) will reproduce. Their fat body cells are rich in RER (for the production of yolk protein precursors). Not much lipids or glycogen accumulate and no protein vesicles. The whole fat body volume is small. At the height of vitellogenesis, it is so small that it is difficult to imagine that it could account for all yolk protein synthesis needed for the large number of eggs (up to about 50–60 per day) the females produce.

When raised in short day conditions (less than 12 hr light per day), reproduction is inhibited and the animals prepare for entering diapause. They start depositing large amounts of reserves in the fat body. Not only lipids and glycogen accumulate but also large amounts of proteins, packed in vesicles. Short day conditions inactivate the *corpora allata*. Thus, it is the absence of JH that causes all these changes. Similar changes take place in the fat body of last instar beetle larvae, a stage in which JH is also absent (unpublished results).

With the insights gained in unraveling the mode of action of absence of JH in pre-metamorphosis (2) the cell biological explanation for the cited effects is probably as follows. As already cited repeatedly, SERCA pumps have a binding site for sesquiterpenoids. As long as the JH titer is high, the SERCA pumps transport Ca^2+^ into the lumen of the RER which causes a secretion of proteins along with Ca^2+^ through the RER–Golgi system. The SERCAs in the SER also pump Ca^2+^ into the lumen of the SER. It is a general rule that SER membranes harbor enzymes for lipid- and steroid biosynthesis (25). As long as the [Ca^2+^] in the SERs lumen is high, these enzymes seem to be inhibited. The result is that no extreme accumulation of lipids in the fat body will occur. When the JH titer drops to zero, Ca^2+^ is no longer pumped into the lumina of both RER and SER. The classical Ca^2+^-stores start releasing part of their stored Ca^2+^. The RER can no longer secrete proteins out of the cell. They remain inside the cell in the form of protein vesicles. In the SER the inhibition of enzymes active in lipogenesis and ecdysteroid synthesis is lifted, resulting in the accumulation of lipid droplets in the fat body and a rising ecdysteroid titer in the hemolymph. All this probably corresponds to early events in the apoptosis pathway.

Thus, as long as the JH titer is high, the apoptosis pathway is inhibited. When the JH titer drops, the apoptosis pathway is induced, resulting in the temporary benefit that willy-nilly reserves are stored that will get a secondary role, beneficial at the organismal level, for remodeling tissues during metamorphosis (13).

We think that the initial steps, namely the roles of a high JH titer and of high farnesol concentrations are probably very similar in inducing obesity in both insects and vertebrates. Morbid lipid deposition in obese humans may reflect some malfunctioning of the FLS/Ca^2+^-ATPase system. A problem is that in vertebrates a situation, in which all FLS can be made totally absent, like this is the case in pre-metamorphosing holometabolous insects, never occurs in life. Whether it can be realized by RNAi is not known. In our opinion, the link FLS-Ca^2+^-obesity deserves further exploration.

## Perspectives for Future Research

The outline of a practical method for analyzing the biosynthetic pathway of FLS (24), in combination with the awareness that this pathway is highly conserved in all eukaryotes, from yeast to mammals, arthropods, and nematodes, opens quite some perspectives for innovative research. It urges for evaluating whether, perhaps this signaling pathway with a causal links to the universal Ca^2+^-homeostasis system might play a key role in maintaining the juvenile state in vertebrates. We hypothesize that the comparison of the pathway in various tissues during successive developmental stages may yield indications whether changes might be causal to state change. Furthermore, a renewed interest in endogenous sesquiterpenoids could, perhaps, contribute to solving some standing problems in medicine.

1. The Hutchinson–Gilford progeria premature aging syndrome has a link to FLS, but its exact mode of action is only partially understood. This type of progeria is a lamin disease, a disease of the nuclear envelope. The cause of this genetic disorder is known. The gene LMNA encodes a protein named prelamin A. Through a process known as prenylation which is not only active in this syndrome, a farnesyl group gets attached to the carboxy-terminus of prelamin A. The farnesyl group allows prelamin to temporarily attach to the nuclear rim. Once the protein is attached, the farnesyl group is removed in normal persons/cells, while in progeria patients it remains attached. The result is that the no longer-farnesylated prelamin, which is now called lamin, does not remain anchored to the nuclear rim. The nucleus displays a normal shape. Failure to remove the farnesyl group permanently affixes the abnormal protein, now called progerin to the nuclear rim. This results in an abnormal shape of the nucleus^2^ (36) (Figure 1).
  It remains unclear how an abnormal shape of the nucleus can cause accelerated aging. There are numerous theories on the causes of aging; for a concise summary see De Loof et al. (19). But if the commonly held view that the nucleus is not an ionically isolated compartment is correct, the form of the nuclear envelope should not matter much. However, if the nucleus has the necessary tools to create its own nucleus-specific environment, a largely overlooked additional level of control of gene expression by inorganic ions (37) emerges. Although the published experimental data are scarce, there is now enough evidence to conclude that the nuclear envelope harbors ion pumps and channels to create a specific intranuclear environment (38–40). The discovery of Ca^2+^-sensitive transcription factors (41) is one of the arguments in favor of the view that the nucleus has its own specific Ca^2+^-homeostasis system (20).
2. With respect to research focused on aging, we think that in the list of nine hall marks of aging (42), two key causes of aging are missing, namely fading cellular electricity (19) and damage due to long lasting excess Ca^2+^ with its causal link to the still poorly understood role of endogenous sesquiterpenoids (2).
3. Alzheimer’s disease: for the moment being (2014) there seems to be some disappointment among researchers that the enormous research effort that has been invested in searching for methods to prevent and cure this very severe disease did not yet yield promising results. A major problem is that the symptoms in humans sometimes become visible about 20 years after the disease started to develop. We think that, perhaps, unraveling the normal mechanisms of being a juvenile may contribute to a better understanding of what can go wrong after the juvenile state ends.
4. Ca^2+^-Homeostasis and the skeleton.

In juveniles, the skeleton of girls is on the average lighter than that of boys. Which elements of the Ca^2+^-homeostasis system are involved and what exactly the role of sex steroids is, remains poorly understood. Osteoporosis, a multifaceted syndrome can become problematic in later life, in particular (but not exclusively) in postmenopausal women. Apparently the system that makes that females extrude more Ca^2+^ from their body than males (20) is already operational from very early in development on until very late in life. Whether manipulating the mevalonate-farnesol pathway may reduce the Ca^2+^-loss may be worth investigating.

### Final conclusion

The evolutionarily ancient and very well conserved biosynthetic pathway leading from mevalonate to farnesol and derivatives is well documented in insects, but in vertebrate endocrinology and physiology hardly any attention has been paid to it. Yet, we think that not only in insects but in vertebrates as well endogenous FLS play a key role in many aspects of development and physiology. Our ideas can be summarized in the – admittedly simplistic – one-liner: “Farnesol/FLS help to smell well, to keep [Ca^2+^]i low and to stay young and slim.”

## Discussion

Time has come to abandon the commonly held view that in vertebrates the juvenile state is automated, meaning that it is purely genetically controlled and that, unlike in insects no specific functional counterpart of a “JH-type signaling substance” is needed. All physiological processes and states of all organisms are controlled one way or another, not necessarily all by hormones.

The theory of evolution states that all contemporary organisms are the progeny of LUCA, the Last Universal Common Ancestor. One should not too readily assume that LUCA was a primitive organism. The environment in which LUCA lived was most probably hostile in several ways. The concentration of Ca^2+^ in the watery environment had probably risen far above the concentration that can hardly be tolerated in the cytosol of contemporary cells, namely about 100 nM. Above that threshold, Ca^2+^ starts changing the conformation and activity of some macromolecules, in particular proteins and chromatin. In other words it becomes toxic. Exactly this property makes that it can act as a secondary messenger in cells, on condition that its concentration rise does not last long. When the rise is more substantial and lasts longer, excess Ca^2+^ can become so toxic that the apoptosis-programed cell death pathway is induced (21). This could be worded as “the Ca^2+^-paradox: intracellularly toxic at low concentrations, but at the organismal level often beneficial at very high concentrations.” This implies that Ca^2+^-homeostasis was already of outmost importance a couple of billion years ago. At least in some species, its underlying mechanisms must have been shaped to near perfection long ago; otherwise “life” would have become extinct. The fact that the structure of Ca^2+^-ATPases of plants, insects, mammals, etc. resembles each other so very well, indicates that the Ca^2+^-ATPases they inherited from LUCA were already shaped to near perfection, and could not undergo substantial changes without becoming less efficient. Another implication is that the way in which the Ca^2+^-homeostasis system is regulated must be very well conserved as well.

All this may sound very logical but it raises the question: What does Ca^2+^-homeostasis and the way it is controlled have to do with being in the juvenile state? It took a touch of serendipity to find the link. It was first found in insects (20), in the following way. Already in Kopeć (43), a Polish biologist and pioneer in insect endocrinology, described that the brain of the moth *Lymantria dispar* is needed for normal development (43). Later, it was found that a pair of tiny glands, the *corpora allata*, which form part of the whole “brain complex,” secrete the hormone “JH.” When these glands are extirpated, precocious metamorphosis is initiated. As cited before, JH turned out to be a simple ester(s) of farnesol. The exact mode of action of JH remained ambiguous until very recently when De Loof et al. (2) re-analyzed the published data. They observed that it had been overlooked that in holometabolous insects the drop to zero of JH titer caused apoptosis in those tissues that had been actively secreting proteins during larval life. The calcium-induced apoptosis paradigm of Orrenius et al. (21) gave the hint that, perhaps, the presence of a high JH titer is required to keep the [Ca^2+^] in the cytosol low. In other words, the juvenile state of insects requires that [Ca^2+^]i is kept low, therefore that the Ca^2+^-pumps keep pumping. The lucky touch of serendipity came when De Loof et al. (2) started searching for known blockers of the Ca^2+^-pump. Evidently, the well-known SERCA blocker thapsigargin showed up. A search for the chemical structure of thapsigargin showed that thapsigargin is, like farnesol and JH, a sesquiterpenoid. Because administration of thapsigargin induces apoptosis in both vertebrates and invertebrates, the conclusion was reached that at least the SERCA pump has a receptor site for sesquiterpenoids. Thus this type of Ca^2+^- pump (to our knowledge no data on PMCAs in the literature) is subject to regulation by endogenous sesquiterpenoids. If the receptor site is promiscuous as suggested by De Loof (20), other lipophilic signaling molecules may compete, in a concentration- and affinity-dependent way in binding to this overlooked type of receptor.

The next step was to evaluate whether the basics of the system that insects use to maintain their juvenile state might also apply to vertebrates. The rationale of this idea was that the control mechanisms for being in the juvenile state have probably been very well conserved since LUCA. This is apparent from the low degree of variability in Ca^2+^-homeostasis systems among plants, vertebrates, invertebrates, etc. The major differences in the role of a FLS among animals are twofold. In insects and nematodes the JH/FLS reaches all cells of the body as a hormone, and, in insects at least its titer in the hemolymph can fall to zero prior to metamorphosis. In vertebrates FLS are synthesized in all cells and throughout life. This corresponds to a mode of action as an “inbrome.” The final effect on Ca^2+^-homeostasis is probably very similar in all eukaryotes.

The concept we outlined here introduces a novel way of thinking, not only on how being a juvenile is regulated, but also on quite some other aspects of evo-devo. It emphasizes that in developmental processes researchers should leave more room for the controlling role of particular inorganic ions, especially Ca^2+^, as well as to the electrical aspects of cell physiology (37, 44, 45). These have come into tribulation by the enormous success of molecular biological techniques that gradually stressed ever more genes and transcription factors.

We are very well aware that our concept is still in the hypothesis and theory phase, and that a lot of additional experimental work is needed before our concept will gain “more body.” The major breakthrough will probably come when the binding site of thapsigargin on the SERCA pump will be chemically defined. The next step would then be to determine the degree of promiscuity of that largely overlooked receptor site.

The advent of the novel method of Rivera-Perez et al. (24) for analyzing which endogenous FLS are synthesized in any tissue via the mevalonate pathway may open new avenues in the numerous fields in which Ca^2+^-homeostasis plays a role, e.g., in metabolism and reproduction (46). The data shown in Table 1 are preliminary and do not exclude that some compounds could have been ingested with the food, but they nevertheless show that FLS are (differentially) omnipresent in the body. The fact that the compounds listed in Table 1 are the same as the ones present in the *corpora allata* of insects, the only tissue that makes them during the larval state, and in nematodes as well (unpublished results) indicates that the mevalonate-FLS biosynthetic pathway is probably as essential in vertebrates as it is in insects and probably all other eukaryotes.

## Conflict of Interest Statement

All authors declare that the research outlined in this paper was conducted in the absence of any commercial or financial relationships that could be construed as a potential conflict of interest.

## Footnotes

^1^http://en.wikipedia.org/wiki/Juvenile_hormone

^2^http://en.wikipedia.org/wiki/Progeria
